# Supplementary material for: Multi‐dataset identification of innovative feature genes and molecular mechanisms in keratoconus
Source: J Cell Mol Med. 2024 Sep 19;28(18):e70079. doi: 10.1111/jcmm.70079 (PMC11412914; doi:10.1111/jcmm.70079)
Supplement: Supplementary file 2 — Material S2. [file JCMM-28-e70079-s002.zip › 7.GSEA analysis/2.ATOH7/KEGG_INTESTINAL_IMMUNE_NETWORK_FOR_IGA_PRODUCTION.html]

Details for gene set KEGG\_INTESTINAL\_IMMUNE\_NETWORK\_FOR\_IGA\_PRODUCTION[GSEA]

|  || Dataset | ATOH7.group.cls#h\_versus\_l |
| Phenotype | group.cls#h\_versus\_l |
| Upregulated in class | h |
| GeneSet | KEGG\_INTESTINAL\_IMMUNE\_NETWORK\_FOR\_IGA\_PRODUCTION |
| Enrichment Score (ES) | 0.51436573 |
| Normalized Enrichment Score (NES) | 1.3462404 |
| Nominal p-value | 0.087890625 |
| FDR q-value | 0.34940928 |
| FWER p-Value | 0.937 |
Table: GSEA Results Summary

  

Fig 1: Enrichment plot: KEGG\_INTESTINAL\_IMMUNE\_NETWORK\_FOR\_IGA\_PRODUCTION      
 Profile of the Running ES Score & Positions of GeneSet Members on the Rank Ordered List

  

| SYMBOL | TITLE | RANK IN GENE LIST | RANK METRIC SCORE | RUNNING ES | CORE ENRICHMENT || 1 | HLA-DQA1 | na | 299 | 0.625 | 0.0568 | Yes |
| 2 | HLA-DPB1 | na | 612 | 0.518 | 0.1020 | Yes |
| 3 | PIGR | na | 712 | 0.496 | 0.1511 | Yes |
| 4 | MADCAM1 | na | 934 | 0.458 | 0.1926 | Yes |
| 5 | CD40 | na | 1048 | 0.440 | 0.2354 | Yes |
| 6 | HLA-DQA2 | na | 1082 | 0.435 | 0.2800 | Yes |
| 7 | CXCR4 | na | 1658 | 0.372 | 0.3023 | Yes |
| 8 | IL10 | na | 2821 | 0.290 | 0.2992 | Yes |
| 9 | TGFB1 | na | 3047 | 0.278 | 0.3218 | Yes |
| 10 | CCL25 | na | 3272 | 0.266 | 0.3432 | Yes |
| 11 | CD40LG | na | 3305 | 0.265 | 0.3700 | Yes |
| 12 | HLA-DMB | na | 3372 | 0.261 | 0.3954 | Yes |
| 13 | CD80 | na | 3860 | 0.242 | 0.4067 | Yes |
| 14 | HLA-DRA | na | 3924 | 0.239 | 0.4299 | Yes |
| 15 | CCL28 | na | 4434 | 0.220 | 0.4382 | Yes |
| 16 | HLA-DQB1 | na | 4600 | 0.215 | 0.4560 | Yes |
| 17 | HLA-DPA1 | na | 4808 | 0.207 | 0.4717 | Yes |
| 18 | CCR9 | na | 4858 | 0.206 | 0.4918 | Yes |
| 19 | MAP3K14 | na | 4980 | 0.202 | 0.5094 | Yes |
| 20 | IL15 | na | 5485 | 0.186 | 0.5144 | Yes |
| 21 | HLA-DRB4 | na | 7427 | 0.132 | 0.4722 | No |
| 22 | CXCL12 | na | 7913 | 0.120 | 0.4709 | No |
| 23 | HLA-DRB5 | na | 8559 | 0.107 | 0.4635 | No |
| 24 | HLA-DRB1 | na | 9300 | 0.094 | 0.4519 | No |
| 25 | TNFSF13 | na | 9317 | 0.093 | 0.4612 | No |
| 26 | TNFRSF17 | na | 10296 | 0.077 | 0.4411 | No |
| 27 | HLA-DOB | na | 10476 | 0.074 | 0.4436 | No |
| 28 | CCL27 | na | 10842 | 0.068 | 0.4403 | No |
| 29 | HLA-DRB3 | na | 11175 | 0.063 | 0.4373 | No |
| 30 | LTBR | na | 11383 | 0.061 | 0.4377 | No |
| 31 | IL6 | na | 11566 | 0.058 | 0.4386 | No |
| 32 | IL2 | na | 13552 | 0.035 | 0.3850 | No |
| 33 | HLA-DMA | na | 14540 | 0.025 | 0.3592 | No |
| 34 | HLA-DOA | na | 15373 | 0.018 | 0.3370 | No |
| 35 | ICOSLG | na | 16605 | 0.008 | 0.3024 | No |
| 36 | ICOS | na | 18396 | -0.008 | 0.2515 | No |
| 37 | IL4 | na | 19594 | -0.018 | 0.2189 | No |
| 38 | IL15RA | na | 22180 | -0.045 | 0.1491 | No |
| 39 | CD28 | na | 24062 | -0.071 | 0.1022 | No |
| 40 | ITGA4 | na | 26362 | -0.112 | 0.0477 | No |
| 41 | ITGB7 | na | 26886 | -0.123 | 0.0454 | No |
| 42 | TNFRSF13C | na | 27097 | -0.128 | 0.0528 | No |
| 43 | CCR10 | na | 27510 | -0.139 | 0.0554 | No |
| 44 | TNFRSF13B | na | 28921 | -0.176 | 0.0332 | No |
| 45 | AICDA | na | 29915 | -0.208 | 0.0263 | No |
| 46 | TNFSF13B | na | 30416 | -0.226 | 0.0355 | No |
| 47 | IL5 | na | 33446 | -0.410 | -0.0090 | No |
| 48 | CD86 | na | 33693 | -0.439 | 0.0298 | No |
Table: GSEA details [plain text format]

  

Fig 2: KEGG\_INTESTINAL\_IMMUNE\_NETWORK\_FOR\_IGA\_PRODUCTION      
 Blue-Pink O' Gram in the Space of the Analyzed GeneSet

  

Fig 3: KEGG\_INTESTINAL\_IMMUNE\_NETWORK\_FOR\_IGA\_PRODUCTION: Random ES distribution      
 Gene set null distribution of ES for **KEGG\_INTESTINAL\_IMMUNE\_NETWORK\_FOR\_IGA\_PRODUCTION**
